# Copper(II) Oxide Nanoparticles Embedded within a PEDOT Matrix for Hydrogen Peroxide Electrochemical Sensing †

**DOI:** 10.3390/s22218252

**Published:** 2022-10-28

**Authors:** Cecilia Lete, Adela-Maria Spinciu, Maria-Gabriela Alexandru, Jose Calderon Moreno, Sorina-Alexandra Leau, Mariana Marin, Diana Visinescu

**Affiliations:** 1 Institute of Physical Chemistry «Ilie Murgulescu» of the Romanian Academy, 060021 Bucharest, Romania; 2 Department of Inorganic Chemistry, Physical Chemistry and Electrochemistry, Faculty of Chemical Engineering and Biotechnologies, University Politehnica of Bucharest, 1-7 Polizu Gh. Street, 011061 Bucharest, Romania; 3 Department of Analytical Chemistry and Environmental Engineering, Faculty of Chemical Engineering and Biotechnologies, University Politehnica of Bucharest, 1-7 Polizu Gh. Street, 011061 Bucharest, Romania

**Keywords:** copper(II) oxide, hydrogen peroxide, conducting polymer, polyol method, sinusoidal voltage method, electrochemical sensor, electroanalysis

## Abstract

The aim of this study is the preparation of nanostructured copper(II) oxide-based materials (CuONPs) through a facile additive-free polyol procedure that consists of the hydrolysis of copper(II) acetate in 1,4-butane diol and its application in hydrogen peroxide sensing. The nonenzymatic electrochemical sensor for hydrogen peroxide determination was constructed by drop casting the CuONP sensing material on top of a glassy carbon electrode (GCE) modified by a layer of poly(3,4-ethylenedioxythiophene) conducting polymer (PEDOT). The PEDOT layer was prepared on GCE using the sinusoidal voltage method. The XRD pattern of the CuONPs reveals the formation of the monoclinic tenorite phase, CuO, with average crystallite sizes of 8.7 nm, while the estimated band gap from UV–vis spectroscopy is of 1.2 eV. The SEM, STEM, and BET analyses show the formation of quasi-prismatic microaggregates of nanoparticles, with dimensions ranging from 1 µm up to ca. 200 µm, with a mesoporous structure. The developed electrochemical sensor exhibited a linear response toward H_2_O_2_ in the concentration range from 0.04 to 10 mM, with a low detection limit of 8.5 μM of H_2_O_2_. Furthermore, the obtained sensor possessed an excellent anti-interference capability in H_2_O_2_ determination in the presence of interfering compounds such as KNO_3_ and KNO_2_.

## 1. Introduction

Copper-based nanoparticles are very attractive for the chemistry of materials due to their interesting physicochemical properties that proved to be relevant in various domains such as (photo)catalysis [1,2], energy storage [3,4,5], electrodes in lithium-ion batteries [6], photodetectors [7], biocidal agents [8,9], and in antitumoral therapy [10,11]. Copper(II) oxide is particularly interesting in electrochemistry due to its nontoxic character, high stability, and semiconducting properties (*p*-type semiconductor), with a narrow band gap (ca. 1.2 eV) and high reactivity in electrocatalytic reactions (one–two electron pathway) at low potential toward clinically relevant analytes such as glucose, uric acid, dopamine, and ascorbic acid [12,13,14,15,16,17,18,19,20]. By taking advantage of soft chemistry approaches, such as hydro-/solvothermal methods [21,22,23,24,25], microwave procedures [26,27,28], and/or polyol-assisted synthesis [2,29,30,31,32], nanoscaled CuOs with diverse morphologies (nano-spheres, nanowires [7,33], rectangular-shaped nanobat- [22], urchin- [24,26], flake-, leaf- or flower-like [28] structures) were obtained. Moreover, copper(II) oxide materials exhibit high specific surface areas and/or hierarchically structures [34] that strongly influence the physicochemical properties and, further, the electrocatalytic activity. Ongoing efforts are nowadays dedicated to achieving new low-cost and biocompatible electroactive copper-based particles with predesigned structural features through facile and eco-friendly solution-based synthesis. Polyols act as mild reducing agents, and thus, reactions carried out in diols or ether diols at temperatures ranging from 130 up to 170 °C afforded mainly Cu_2_O and only rarely CuO [2,29,30]. In addition, the reaction time/temperature, concentration of metal precursors, or the chain length of the polyalcohol proved to influence the morphostructural characteristics and composition of the oxidic material [35,36,37].

Hydrogen peroxide (H_2_O_2_) plays essential roles in pharmaceutical, clinical, food, environmental, and textile applications, as well as in different biological processes (activation of immune cells, vascular remodelling, apoptosis, stomatal closure, and root growth), justifying the upsurge in need for new analytical devices for H_2_O_2_ quantification. As a short-lived compound, H_2_O_2_ is transformed into H_2_O and O_2_ in the presence of enzymes such as catalase and peroxidase and becomes toxic to cells when it exceeds 50 µM [38]. Generally, its toxicity is related to highly reactive hydroxyl radicals formed by UV radiation exposure [39] or Fenton reactions occurring in the presence of transition metals [40]. Moreover, H_2_O_2_ can determine the liberation of irons from hem proteins which will be involved subsequently in Fenton reactions. Considering the great impact on human health, the FDA has established a limit of 0.5 ppm of H_2_O_2_ (14.7 µM) in finished food products. Hydrogen peroxide is an electroactive molecule, and thus, the development of simple, rapid, sensitive, and cost-effective electrochemical sensors for the efficient and accurate detection of H_2_O_2_ is of great interest [41]. In the search for cheap materials and facile synthetic procedures, electrodes modified with copper oxide-based particles were more and more involved in the nonenzymatic sensing of hydrogen peroxide [25,42,43,44,45,46,47,48]. For example, glassy carbon electrodes (GCE) modified with electrodeposited nanosheets of Cu@CuO [42], hollow [45], 3D flower-like CuO/Cu [47], or nest-like CuO nanostructures [25] proved to have excellent electrochemical catalytic performances towards H_2_O_2_ reduction in terms of sensitivity, a wide linear response range, a low detection limit, and/or fast responses. In addition, when used as a direct electrode material, CuO deposited on Cu/Ni foams showed rapid and selective responses to hydrogen peroxides [43,44].

Herein, we report on the facile and direct obtaining of copper(II) oxide nanoparticles (denoted as CuONPs) through an additive-free polyol procedure. The CuONP material was further employed in the assembly of a modified GCE/PEDOT/CuONP electrode in which electrocatalytic activity toward hydrogen peroxide was probed. The PEDOT coating was electrodeposited onto a GCE surface by means of the sinusoidal voltage method according to the procedure described in the experimental section [49,50,51]. The obtained sensor was tested for H_2_O_2_ determination, and its analytical performance was evaluated.

## 2. Materials and Methods

### 2.1. Chemicals

Copper acetate Cu(CH_3_COO)_2_·H_2_O (Sigma-Aldrich, St. Louis, MO, USA) and 1,4-butanediol [HO(CH_2_)_4_OH, 1,4-BD] (Sigma-Aldrich) of analytical grade were used without further purification. Compounds including 3,4-ethylenedioxythiophene (EDOT, Sigma Aldrich), potassium nitrate (Sigma-Aldrich), potassium nitrite (Sigma-Aldrich), sodium chloride (Sigma-Aldrich), hydrogen peroxide 30% (*w*/*w*) (Sigma-Aldrich), potassium hexacyanoferrate (III) (Sigma-Aldrich), sodium hexacyanoferrate (II) (Sigma-Aldrich), monobasic potassium phosphate (Sigma-Aldrich), and dibasic potassium phosphate (Sigma-Aldrich) were of analytical grade. Stock hydrogen peroxide solutions were prepared daily with double-distilled water.

### 2.2. Synthesis of the Copper-Based Material

The copper oxide material was obtained by hydrolysis of copper(II) acetate (0.5 M) in 1,4-butandiol, at 140 °C, which afforded dark-brown precipitates. In a typical synthesis, a solution of 1.9965 g of Cu(CH_3_COO)_2_·H_2_O in 20 mL of 1,4-butanediol was heated under stirring at the reaction temperature in a round-bottom flask fitted with a reflux column for 6 h. The precipitation of a dark-brown CuONP material occurred after two hours. After cooling at room temperature, the precipitate was collected by centrifugation and washed with ethanol.

### 2.3. Preparation of the CuONP-Based Electrochemical Sensor

The CuONP-based electrochemical sensor was prepared by using the following procedures:

(A) Drop casting technique: Ethanol 98% solutions containing selected CuO nanoparticle concentrations of 1, 3, and 5 mg/mL were prepared. The freshly prepared solutions were sonicated for 30 min. Then, a known volume of 4 µL of CuO-containing solution was dropped onto the electrode surface. The electrode was left to dry in the laboratory for 4 h. The obtained sensor was denoted as GCE/CuONPs;
(B) Sinusoidal voltage deposition of the PEDOT coating onto naked GCE followed by deposition of CuO nanoparticles using the drop casting technique. The obtained sensor was denoted as GCE/PEDOT-CuONPs;
(C) Drop casting of CuO nanoparticles onto naked GCE and electrodeposition of the PEDOT coating over the modified electrode surface using the sinusoidal voltage procedure. The obtained sensor was denoted as GCE/CuONPs/PEDOT.

The sinusoidal voltage procedure consists of the application of a sinusoidal excitation voltage with a fixed frequency of 50 mHz and an amplitude of 0.25 V (rms) over a constant potential of +0.60 V. The deposition time was 20 min. The electrodeposition of the PEDOT coating using the sinusoidal voltage procedure was carried out from an aqueous solution containing 10 mM PEDOT monomer and 0.1 M KNO_3_.

**Scheme 1:**
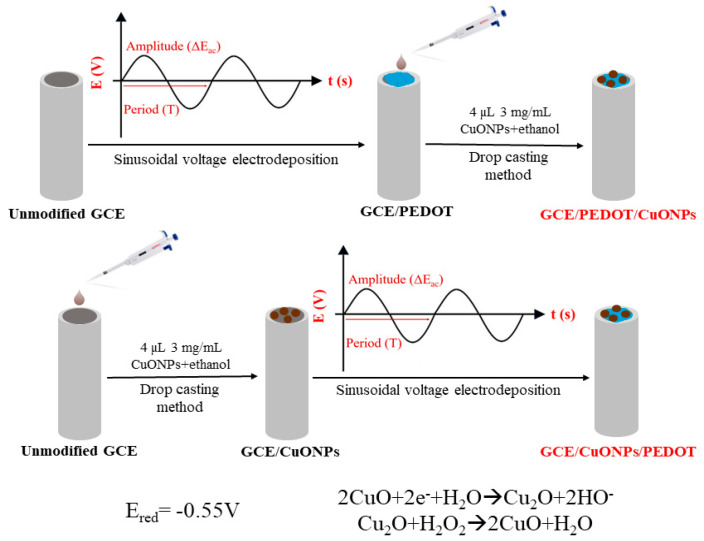
Schematic pathways of preparing the CuONP-based electrochemical sensor.

Scheme 1 illustrates the main steps for the set-up of the CuONP-based electrochemical sensor.

### 2.4. Apparatus and Measurements

*Characterization*. The Fourier transform infrared (FTIR) spectrum (KBr pellets) was recorded with a FTIR Bruker TENSOR V-37 spectrophotometer. UV–vis measurements were performed on a PerkinElmer Lambda-35 (200–1100 nm) spectrophotometer. The structure of the resulting powders was examined at room temperature with a SHIMADZU XRD 6000 diffractometer (Shimadzu Corporation, Kyoto, Japan) using Ni-filtered CuKα radiation (λ = 1.5418 Å) with a scan step of 0.02° and a counting time of 1 s/step for 2*θ* ranging between (20–80°). The average crystallite size (D) of the samples was determined using the Williamson−Hall equation, *β*_hkl_cos *θ*_hkl_ = kλ/D + 4εsin*θ*_hkl_, where λ is the wavelength of the CuKα radiation, k is a constant equal to 0.9, and *β*_hkl_ is the instrumental-corrected broadening measured at the half-maximum intensity of the (hkl) peak at the *θ*_hkl_ Bragg diffraction angle. The morphology of the particles was evaluated by scanning electron microscopy (SEM), and the SEM measurements were carried out on a Quanta FEG microscope. The morphological properties of the obtained CuONPs, including their shape, internal structure, and spatial organization, were investigated through the HR-STEM technique using a Hitachi HD-2700 instrument operating at a 200 kV accelerating voltage equipped with an energy dispersive X-ray (EDX) Oxford Instruments X-max 100 TLE detector. Two kinds of electron images were acquired simultaneously at the same magnification and in the same location on the sample by using two different imaging techniques: the high angle annular dark field technique for ZC–phase contrast imaging and the bright field technique for transmission electron imaging.

The electrochemical measurements were performed using a potentiostat/galvanostat Autolab 302N (Ecochemie, Kanaalweg, The Netherlands) connected via an interface to a personal computer and controlled by means of GPES and FRA software. All electrochemical experiments were carried out using an undivided electrochemical cell (Metrohm, Herisau, Switzerland) containing a glassy carbon disk electrode (GCE, diameter of 3 mm; Metrohm), a glassy carbon rod (Metrohm), and silver/silver chloride (Ag/AgCl/KCl 3M) (Metrohm) as working, auxiliary, and reference electrodes, respectively. The glassy carbon disk working electrode was cleaned prior to measurements by mechanical polishing with alumina powders with diameters of 0.3 and 0.05 μm, followed by rinsing with double-distilled water. Afterwards, an electrochemical cleaning protocol that consists of the potential cycling of the GCE in 0.5 M H_2_SO_4_ aqueous solution between (−1.0) V and +1.0 V at a potential scan rate of 100 mV/s for 20 scans was applied. The aqueous solutions were bubbled with argon 5.0 (Linde, Timișoara, Romania), and an argon blanket was maintained over the solutions during the measurements. The electrochemical impedance measurements were carried out by means of the FRA2 module in an aqueous solution containing 5 mM Na_4_[Fe(CN)_6_]/K_3_[Fe(CN)_6_] and 0.5 M KNO_3_ using a frequency range from 10 kHz to 0.05 Hz and a 10 mV (rms) amplitude of the sin wave at the bias potential of 0.22 V (the open circuit potential). All measurements were performed at room temperature (25 °C).

### 2.5. Analytical Protocols

The analytical performance of the prepared sensors was investigated using cyclic voltammetry and chronoamperometry. The detection of H_2_O_2_ was carried out in 0.1 M NaOH aqueous solution using chronoamperometry at a working potential of (−0.4) V in the concentration range between 0.04 and 10 mM. The real sample analysis was performed using cyclic voltammetry and standard addition protocols. Milk samples were diluted with a 0.1 M NaOH electrolyte solution in a 1:20 volume ratio, and the cyclic voltammogram was registered. Afterwards, the sample was spiked with known amounts of H_2_O_2_, and the cyclic voltammograms were recorded after each addition. The cathodic peak current measured at −0.45 V was used in the calculation of the H_2_O_2_ content and the recovery values.

## 3. Results

Copper(II) oxide is rarely obtained in polyol-assisted reactions, the hydrolysis of copper(II) precursors in diols/ether diols (as reduction agents) affording copper(I) oxide, and/or metallic copper particles. The long-chain diol 1,4-BD and mild reaction conditions (140 °C) are likely to be the cause of the unusual stabilization of CuO [36].

### 3.1. PhysicoChemical Characterization of the CuONP Material

#### 3.1.1. FTIR and UV–Vis Spectroscopy

**Figure 1:**
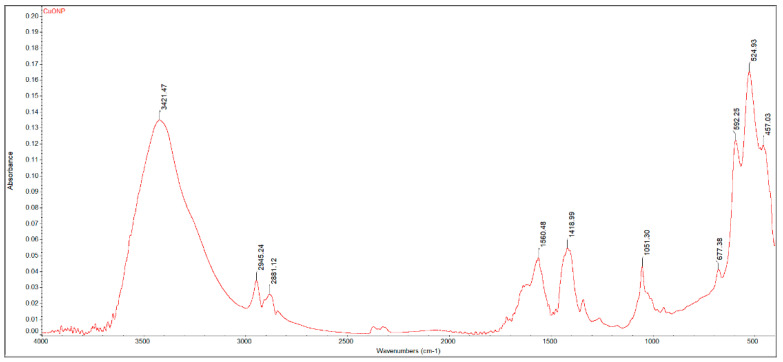
FTIR spectrum of the CuONP material.

The FTIR spectrum of the copper-based sample (Figure 1) shows an intense, broad, and structured absorption in the 400–700 cm^−1^ region, attributed to the asymmetric (457 cm^−1^) and symmetric (525, 592 cm^−1^) stretching vibration of the Cu^II^-O bond, as well as of the O-Cu^II^-O asymmetric vibration (677 cm^−1^) [52]. Traces of water (ν_O-H_ at 3400/1655 cm^−1^), unreacted diol (ν_C-OH_ at 1052 cm^−1^; ν_C-H_ at 2880/2945 cm^−1^), or acetate groups (ν_sym/asym_COO^−^ at 1720/1419 cm^−1^) were also identified in the FTIR spectrum of the dark-brown precipitate. Since no post-treatment calcination was carried out, the byproducts were likely adsorbed on the surface of the material.

**Figure 2:**
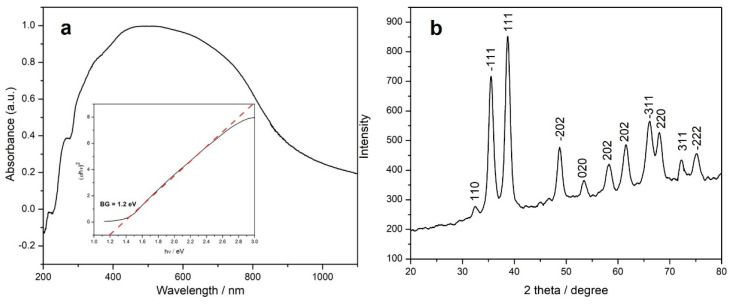
UV–vis spectrum (**a**) and X-ray powder diffractogram (**b**) of the CuONP sample (inset Figure 1a: Tauc plot for the estimation of the energy band gap of the CuONP oxide).

The electronic spectrum of the sample is represented in Figure 2a and shows a broad band centered at ca. 500 nm, characteristic for the square-planar {CuO_4_} chromophore within the copper(II) oxide lattice. The weak shoulders in the UV region (218 and 260 nm) can be assigned to the organic species adsorbed onto the CuO surface. The band gap (BG) of the copper-based sample was determined from the absorption values using the Tauc plot [53], the estimated band gap being 1.2 eV (see inset of Figure 2a).

#### 3.1.2. XRD Analysis

The XRD pattern of the copper-based sample is represented in Figure 2b, the peaks being indexed to the monoclinic structure of tenorite (copper oxide, JCPDS PDF, no. 05-0661) with the following cell parameters: *a* = 4.701(3), *b* = 3.430(2), *c* = 5.135(5) Å, *β* = 99.51(1)°, and *V* = 81.66 Å^3^. The broad peaks could be related to the small dimensions of the particles. The mean crystallite size value is ca. 8.7 nm with a lattice strain of 1.14.

#### 3.1.3. Microstructural Characterization

**Figure 3:**
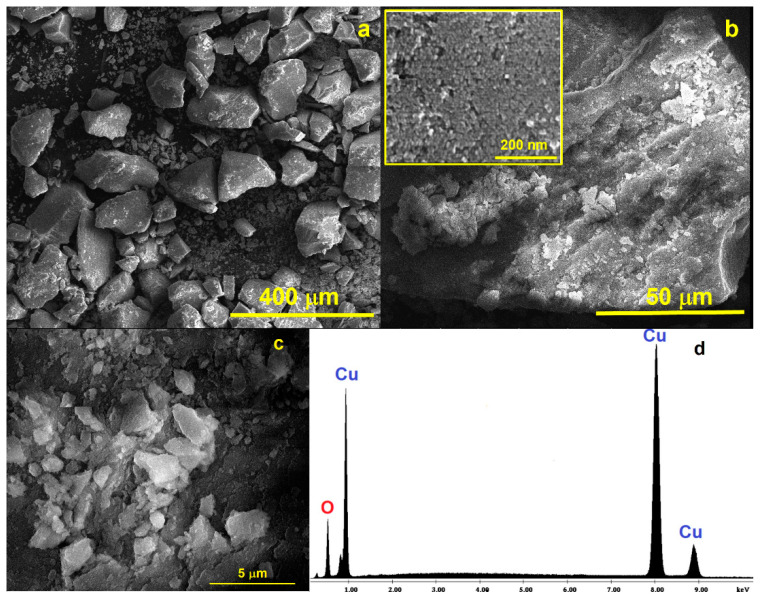
SEM micrographs of the CuONP material at different magnifications: (**a**) 400 μm; (**b**) 50 μm (inset: details of one aggregate at 200 nm showing the spherical nanosized CuONP particles); (**c**) 5 μm; and (**d**) EDAX spectrum for the CuONP material.

The SEM micrographs represented in Figure 3 show the formation of large and quasi-prismatic aggregates of CuO nanoparticles with different sizes ranging from (100 nm up to ca. 200 μm). The inset of Figure 3b depicts details of one aggregate showing the constituent small spherical particles of the CuONPs gathered in a larger structure.

**Figure 4:**
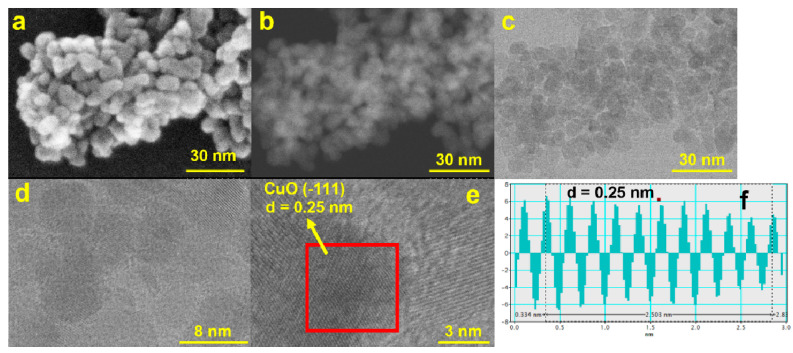
STEM micrographs for the CuONP material using (**a**) SE—secondary electrons, (**b**) ZC—phase contrast, and (**c**) TE—transmission electron detectors; (**d**) HR-STEM image at ×4000 K and (**e**) ×8000 K magnification and the corresponding profile for d-spacing measurements (**f**).

The obtained EDX results confirm that only Cu and O atoms are present in the prepared nanoparticles (Figure 3d). Scanning transmission electron microscope (STEM) measurements were also performed to achieve the ultrahigh-resolution images (UHR-STEM) for the CuONP material (Figure 4). The STEM micrographs confirm the formation of spherical particles for CuONPs of ca. 8 nm, values quite close to those estimated from the XRD data. HR-STEM images acquired at ×4000 and ×8000 k magnification (Figure 4d,e) prove the spherical shape and crystallinity of the CuONPs. In addition, 3D profile measurements were performed (Figure 4f), the interplanar distance of 0.25 nm being in line with the d value of the monoclinic CuO crystal plane (−111).

The BET analysis proved that the CuONP material is mesoporous, with an *S*_BET_ value of 82.94 m^2^/g and a total pore volume of 0.135 cm^3^g^−1^, with irregular nanopores of several nanometers. The formation of hierarchically structured oxide materials is a common characteristic in syntheses carried out in additive-free 1,4-BD [36,54].

### 3.2. Electrochemical Characterization of CuONP-Based Sensors

**Figure 5:**
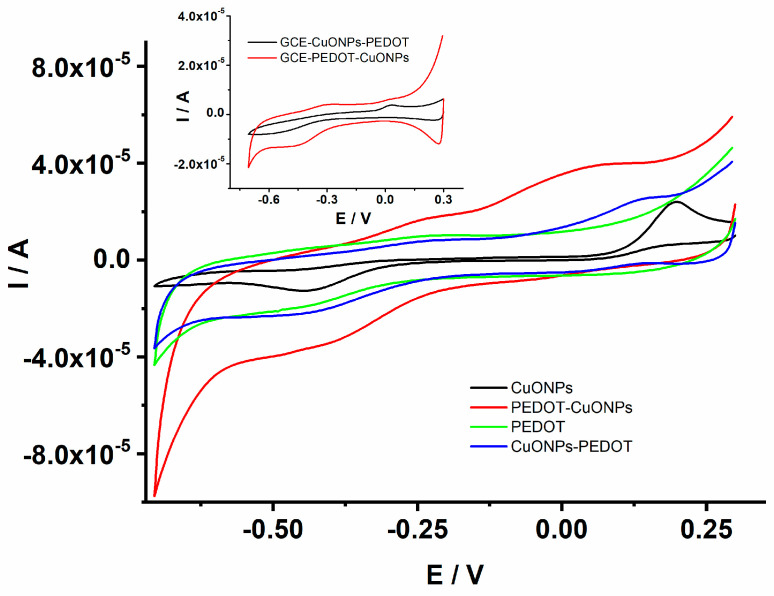
CV traces recorded for the GCE/CuONPs, GCE/PEDOT-CuONPs, GCE/PEDOT, and GCE/CuONPs-PEDOT electrodes in 0.1 M NaOH containing 1 mM H_2_O_2_; potential scan rate: 50 mV/s. Inset: CV traces for the GCE/CuONPs-PEDOT and GCE/PEDOT-CuONPs electrodes.

The electrochemical properties and the electrocatalytic activity of the CuONP-based sensors were investigated using cyclic voltammetry (CV) and electrochemical impedance spectroscopy (EIS) in an aqueous solution containing a redox probe as well as 0.1 M NaOH aqueous solutions. First, the investigation of the prepared sensors was carried out in 0.1 M NaOH to assess the electrocatalytic performance. Figure 5 depicts the CVs recorded for the GCE/CuONPs, GCE/PEDOT, GCE/CuONPs-PEDOT, and GCE/PEDOT-CuONPs sensors in the presence of 1 mM H_2_O_2_. It can be noticed that the GCE/PEDOT electrode displayed a CV with a well-known characteristic of a conducting polymer as expected, i.e., a quasi-rectangular shape specific to capacitive behavior is observed due to the presence of the PEDOT layer on the electrode surface, while there is a very small cathodic peak related to the H_2_O_2_ analyte at ca. (−0.45) V. On the contrary, both the GCE/CuONPs and GCE/PEDOT-CuONPs sensors displayed one enhanced cathodic peak at (−0.45) V and an anodic peak at ca. 0.19 V for CuONPs and at 0.04 V for a PEDOT-CuONPs composite material, respectively. The cathodic peak located at (−0.45) V can be assigned to the H_2_O_2_ reduction under these experimental conditions. Compared to the CV trace recorded for the PEDOT coating, it could be inferred that the enhanced cathodic peaks observed by the GCE/CuONPs and GCE/PEDOT-CuONPs sensors can be ascribed to the CuONPs’ electrocatalytic activity towards H_2_O_2_ reduction. The use of the PEDOT coating was intended primarily to ensure a favorable (stable) microenvironment for the CuONPs. The increase in the cathodic current at (−0.45) V for the GCE/PEDOT-CuONPs compared to the GCE/CuONPs can be related to a better inclusion and distribution of CuONPs within the PEDOT layer which favours the availability of the multifaceted CuONPs to the chemical reaction with the H_2_O_2_ analyte. These results clearly demonstrate the electrocatalytic activity of CuONPs towards H_2_O_2_ reduction. The sensor with a geometry consisting of overlaying CuONPs drop coated onto the GCE surface with a PEDOT layer exhibited a lower reduction peak current for H_2_O_2_, as can be observed in the inset of Figure 5. This can be due to the hindrance of the PEDOT layer on the H_2_O_2_ diffusion towards the active centers of the CuONPs. The PEDOT coatings were electrodeposited in both sensors’ geometries by using the sinusoidal voltage procedure which ensures an increased porosity and roughness of the polymeric layer compared to classical electrochemical methods, such as potentiostatic and galvanostatic ones. However, the CuONPs/PEDOT geometry does not display better electrocatalytic activity compared to the PEDOT-CuONPs one. The PEDOT-CuONP-based electrode displays a cathodic peak at −0.45 V and an anodic one at 0.04 V, respectively, towards H_2_O_2_, and a larger increase in the current could be observed. The CuONPs-PEDOT-based electrode displays a similar redox behavior, but with a lower current compared to the PEDOT-CuONPs electrode. On the contrary, the PEDOT itself shows almost no redox wave for H_2_O_2_. Therefore, the PEDOT-CuONP-based sensor displayed the best catalytic effect compared to the other composite electrodes.

Consequently, the investigation was further focused on revealing the electrochemical properties and analytical performance of the PEDOT-CuONPs’ geometry by controlling the loading of CuONPs onto the PEDOT matrix.

**Figure 6:**
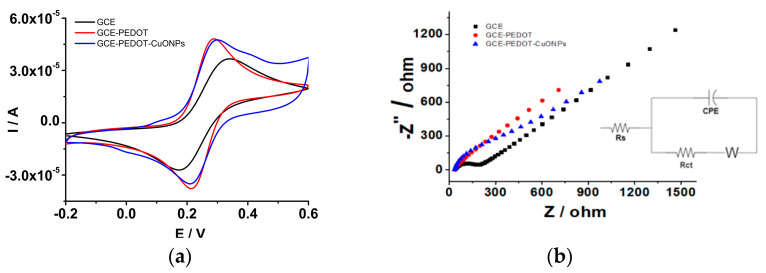
(**a**) CVs recorded for the naked GCE (black line), GCE/PEDOT (red line), and GCE/PEDOT-CuONPs (blue line) in an aqueous solution containing 5 mM Na_4_[Fe(CN)_6_] and 0.5 M KNO_3_. Potential scan rate: 50 mV/s. (**b**) Electrochemical impedance spectra recorded for the naked GCE (full rectangles), GCE/PEDOT (full circles), and GCE/PEDOT-CuONPs (full triangles) in an aqueous solution containing 5 mM Na_4_[Fe(CN)_6_]/K_3_[Fe(CN)_6_] and 0.5 M KNO_3_. Inset: the electrical equivalent circuit used in the fitting of the EIS data.

In the first step, the electrochemical characterization of the PEDOT coating was performed to ensure a reliable and favourable matrix for the CuONPs’ subsequent deposition. For this purpose, the PEDOT coating was electrodeposited onto the GCE surface by means of the sinusoidal voltage method according to the procedure described in the experimental section. Afterwards, the GCE/PEDOT-modified electrode was investigated in an aqueous solution in the presence of a redox probe. For comparison, the naked GCE was also investigated. The CV measurements were carried out in a solution containing an Na_4_[Fe(CN)_6_] redox probe at different potential scan rates in order to elucidate the interfacial properties of the developed sensors. Figure 6a depicts the CVs recorded in an aqueous solution containing 5 mM Na_4_[Fe(CN)_6_] and 0.5 M KNO_3_. The GCE/PEDOT-modified electrode displayed higher anodic and cathodic peaks compared to the naked GCE. In addition, the peak-to-peak potential separation (Δ*E_p_* = *E_pa_* − *E_pc_*) observed for GCE/PEDOT (71 mV) is smaller than that for GCE (165 mV). The rising part of the voltammograms for the GCE/PEDOT-modified electrode is steeper than that observed at the GCE, attesting to the presence of a porous layer on the electrode surface. The CV trace for GCE/PEDOT-CuONPs is characterized also by a smaller peak-to-peak potential separation (82 mV), attesting to a fast electron transfer at the electrode/solution interface of the redox probe. These results demonstrate the improved electron transfer capability of the PEDOT and PEDOT-CuONPs coatings compared to the unmodified GCE. The enhanced electron transfer capability is a prerequisite for developing fast and sensitive electrochemical sensors. Therefore, the benefits of the PEDOT coating as a favourable microenvironment for CuONPs’ subsequent deposition are clearly demonstrated. A good correlation between the electrochemical impedance spectroscopy (depicted in Figure 6b) and cyclic voltammetry data can be observed in Figure 6a. The EIS for the GCE displays the characteristic shape for a diffusion-controlled electron transfer process, with a semicircle at high frequencies related to the electron transfer followed by a straight line at low frequencies ascribed to the diffusion of the redox probe. There is a noticeable charge transfer resistance of ca. 278.5 Ω in the case of the GCE. On the contrary, the EIS recorded at the GCE/PEDOT displayed a linear region at low frequencies, while the semicircle at high frequencies is very small, with a charge transfer resistance of 69.8 Ω, attesting to the improved electron transfer capability of the PEDOT coating. Moreover, the EIS spectrum of the GCE/PEDOT-CuONPs displays the characteristics of the Randles circuit model, with low charge transfer resistance of 60.4 Ω, comparable to the GCE/PEDOT electrode.

The EIS spectra were fitted using an electrical circuit (see inset of Figure 6b) consisting of the solution resistance (*R_s_*), the charge transfer resistance (*R_ct_*), the infinite-length Warburg diffusion impedance (*Z_W_*), and the constant phase element (CPE, Q). For instance, the following fitted values for the GCE/PEDOT-CuONPs sensor were obtained: *R_s_* = 49.1 Ω, *R_ct_* = 60.4 Ω, *Q/Y*_0_ = 2.5 × 10^−4^ Ω^−1^ s^−n^, *n* = 0.85, and *Z_W_* = 2.4 × 10^−2^ Ω.

These results pointed out the feasibility of the CuONP sensing element in the development of an electrochemical sensor for H_2_O_2_ detection.

**Figure 7:**
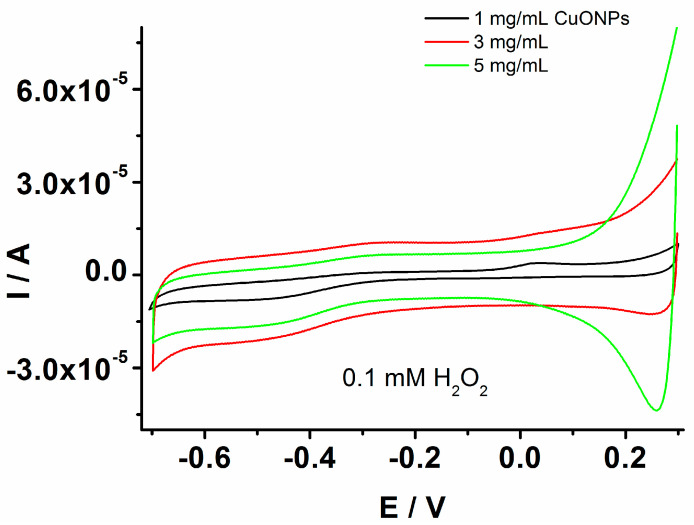
CVs recorded for the GCE modified with the composite material based on PEDOT-CuONPs with different loadings of CuONPs on the PEDOT matrix, 1 mg/mL (black line), 3 mg/mL (red line), and 5 mg/mL (green line), in the presence of 0.1 mM H_2_O_2_; potential scan rate: 50 mV/s.

The results endowed the investigation of the GCE/PEDOT-CuONPs sensor’s geometry by controlling the loading of CuONPs to optimize the electrocatalytic activity and analytical performance of the developed sensors. In this sense, the use of various loadings of CuONPs on the PEDOT matrix was investigated in the sensor’s optimization process. The CuONP loadings were controlled by the deposition of the same volume of nanoparticle solutions onto the electrode surface but varying the solution concentrations in the range of 1–5 mg/mL. These concentration values were selected considering previously published results in the literature [45] and preliminary data obtained by experimental investigation. Figure 7 displays the cyclic voltammograms recorded for the GCE modified by the composite material prepared with different CuONP concentrations in the presence of 0.1 mM H_2_O_2_. The best response of the GCE/PEDOT-CuONPs toward H_2_O_2_ reduction was for a 3 mg/mL CuONP concentration.

The electrochemical active surface area of the CuONP-based sensor is an important parameter that exerts a great influence on the analytical performance and enables the proper selection of the best modifying layer and sensor’s geometry. Consequently, the electrochemical active surface area was estimated for GCE/PEDOT-CuONPs sensors with various CuONP loadings using the redox probe, Na_4_Fe(CN)_6_, by means of the cyclic voltammetry technique and the Randles–Sevcik Equation (1) at 25 °C [55]:

i_p_ = 2.69 × 10^5^ n^3/2^ A D^1/2^ C v^1/2^ (1)

where i_p_ is the peak current (A), n is the number of transferred electrons (n = 1), A is the geometric surface area (cm^2^), D is the diffusion coefficient of the redox probe (6.67 × 10^−6^ cm^2^/s), C is the concentration of the redox probe (mol/cm^3^), and v is the potential scan rate (V/s). The electrochemical surface area could be estimated from the slope of the plot of the peak current dependence versus the square root of the potential scan rate. The obtained values for the GCE/PEDOT-CuONPs sensor containing 1, 3, and 5 mg/mL CuONP loadings were 0.082, 0.110, and 0.485 cm^2^, demonstrating the benefits of CuONPs in improving the electron transfer capability. An enhanced electrochemical surface area is beneficial for analytical applications by improving the sensitivity of the measurements.

**Figure 8:**
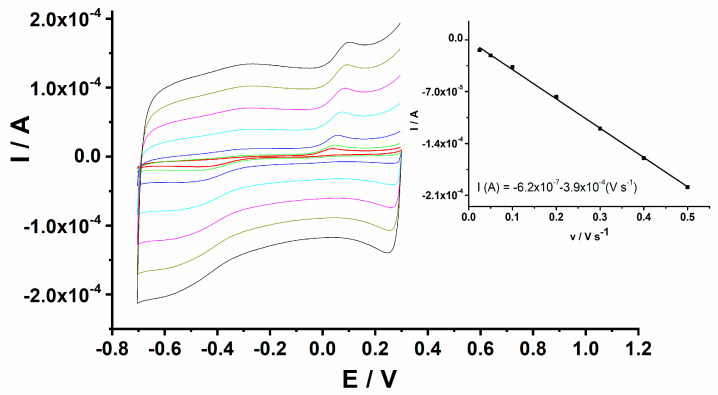
CVs for GCE/PEDOT-CuONPs in 0.1 M NaOH containing 1 mM H_2_O_2_ recorded at various potential scan rates of 0.025, 0.05, 0.1, 0.2, 0.3, 0.4, and 0.5 V s^−1^. Inset: linear dependence of the cathodic peak current versus the potential scan rate.

The electrochemical behaviour of H_2_O_2_ at the GCE/PEDOT-CuONP-based sensor was investigated in a 0.1 M NaOH solution containing 1 mM H_2_O_2_ using cyclic voltammetry at different potential scan rates ranging from 25 to 500 mV/s, and the corresponding CV traces are depicted in Figure 8. The linear dependence of the cathodic peak current on the potential scan rate suggests a surface-controlled process in the case of H_2_O_2_ reduction at the electrode/solution interface (inset of Figure 8).

The dependence of the cathodic peak potential (E_p_ = −0.45 V) on the logarithm of the potential scan rate (ν) for the irreversible electrochemical reduction of H_2_O_2_ is linear according to Laviron’s Equation (2) [56]:

E_p_ = E^0^ + 2.3 RT/(αnF) log(RTk^0^_f_)/(αnF) + 2.3 RT/(αnF) log (ν) (2)

where E_p_ is the reduction peak potential, E⁰ is the formal potential, n is the electron number involved in the oxidation/reduction process, K_f_^0^ is the electrochemical rate constant, α is the transfer coefficient, T is the temperature (298 K), R is the general gas constant (8.314 J mol^−1^ K^−1^), and F is the Faraday constant (96,485 C mol^−1^). For a totally irreversible electrode process, α is assumed to be 0.5. Thus, the value of αn can be easily calculated from the slope of the E_p_ versus log ν plot. The calculated number of electrons from the slope of the linear regression equation was 2 (linear equation: E_p_ = −0.54 − 0.068 log ν).

### 3.3. Analytical Applications of CuONP-Based Sensors

**Figure 9:**
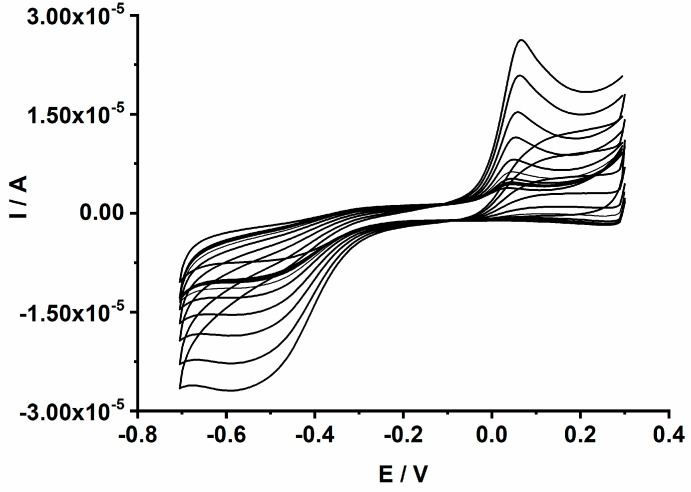
CVs recorded for the GCE/PEDOT-CuONPs electrochemical sensor in 0.1 M NaOH aqueous solutions containing different H_2_O_2_ concentrations ranging from 0.04 to 2 mM. Potential scan rate: 50 mV/s.

The electrocatalytic and analytical performances of the CuONP sensing material were investigated in an aqueous solution containing 0.1 M NaOH. Various sensors’ geometries, including GCE/CuONPs, GCE/CuONPs-PEDOT, and GCE/PEDOT-CuONPs, were systematically investigated to elucidate the benefits of the polymeric matrix and the synergy between the CuONPs and the PEDOT coating. The GCE/PEDOT-CuONPs sensor displayed the best analytical response toward H_2_O_2_ compared to the other CuONP-based sensors (see Figure 5). The cyclic voltammograms recorded for the GCE/PEDOT-CuONPs sensor in an aqueous solution containing 0.1 M NaOH and different H_2_O_2_ concentrations are displayed in Figure 9. The electrocatalytic activity of the CuONPs is demonstrated by the presence of a cathodic peak located at ca. (−0.55) V, which is ascribed to the reduction of Cu(II) to Cu (I), and an anodic peak at ca. +0.12 V that corresponds to the oxidation of Cu(I) to Cu(II). Cu(I) is involved in the chemical reaction of H_2_O_2_ reduction according to the following proposed mechanism [45]:

2CuO + 2 e^−^ + H_2_O → Cu_2_O + 2HO^−^ (3)

Cu_2_O + H_2_O_2_ → 2CuO + H_2_O (4)

**Figure 10:**
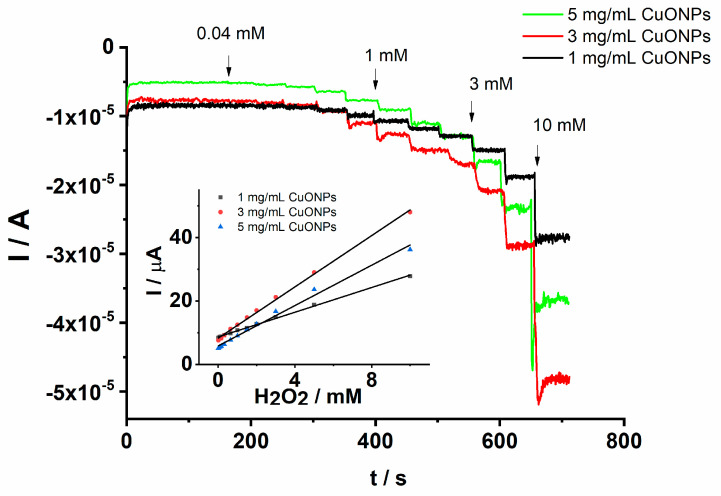
Chronoamperograms recorded for the GCE/PEDOT-CuONPs electrochemical sensor with different CuONP loadings in an aqueous solution containing 0.1 M NaOH and added H_2_O_2_ concentrations of 0.04, 0.08, 0.16, 0.32, 0.64, 1, 1.5, 2, 3, 5, and 10 mM. Working detection potential: (−0.4) V. Inset: the corresponding calibration plots.

The cathodic peak current increases linearly with the H_2_O_2_ concentration over the range from 0.04 to 2 mM. The linear dependence of the cathodic peak current on the H_2_O_2_ concentration is explored in the analytical applications constituting the main criterion in the development of electrochemical sensors. The analytical performance of the proposed GCE/PEDOT-CuONPs sensor was further investigated by means of chronoamperometry. It is well known that chronoamperometry is a very sensitive electroanalytical technique used in the optimization of electrochemical sensors and biosensors. Figure 10 displays the chronoamperograms recorded for the GCE/PEDOT-CuONPs sensor with different CuONP loadings (1, 3, and 5 mg/mL) in a 0.1 M NaOH solution containing H_2_O_2_ concentrations ranging between 0.04 and 10 mM.

It can be observed that there is a fast response of the amperometric sensor after the addition of each H_2_O_2_ amount at the working potential of (−0.4) V. The response time is less than 2 s, demonstrating a fast analytical response and improved electrocatalytic efficiency due to the CuONPs. The current increases linearly with the H_2_O_2_ concentration in the range of 0.04 to 10 mM. These results demonstrate the good capability of the CuONPs in the electroanalytical detection of H_2_O_2_ under dynamic conditions. The corresponding calibration plots obtained from the chronoamperograms are shown in the inset of the Figure 10. It can be remarked that the response of the electrochemical sensor prepared with 3 mg/mL CuONPs is the best as in the case when cyclic voltammetry was used in the determination of H_2_O_2_. Despite an electroactive surface area higher for the prepared sensor with 5 mg/mL CuONPs, the sensitivity (3.84 µA/mM) was lower than in the case when a 3 mg/mL CuONP concentration (4.60 µA/mM) was used in the development of the sensor. For instance, the obtained linear regression equation for 3 mg/mL CuONPs is I (µA) = −7.84–4.60 [H_2_O_2_] mM and r = 0.9985, attesting to a good correlation of the peak current on the analyte concentration. The lower sensitivity in the case of the 5 mg/mL CuONP concentration could be assigned to the higher thickness of the PEDOT-CuONPs coating which can hinder the electron transfer process at the electrode–electrolyte interface. On the other hand, it is well known that thinner films are more sensitive and stable.

The detection limit of the GCE/PEDOT-CuONPs sensor was estimated using the following criterion: *3s*/*m*, where *s* is the standard deviation of the blank and *m* is the slope of the calibration plot. Under these experimental conditions, a detection limit of 8.5 μM was obtained. The low detection limit and the wide linear response range are comparable or even better than the other sensors previously published in the literature and attest to the good analytical performance of the proposed GCE/PEDOT-CuONPs sensor (see Table 1). Another important analytical parameter, the working detection potential, is close to or less negative than some previously reported in the literature.

**Figure 11:**
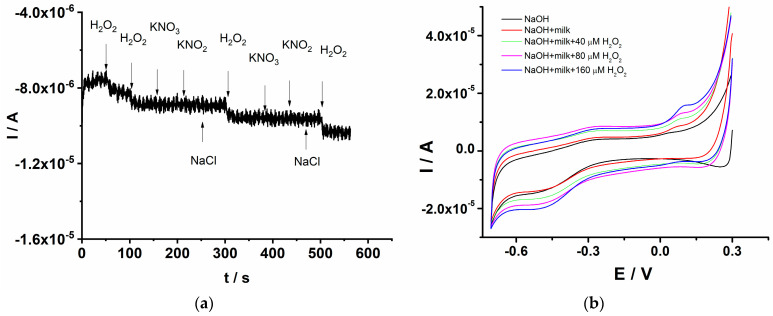
(**a**) Chronoamperogram for H_2_O_2_ selective detection for the GCE/PEDOT-CuONPs sensor in the presence of various interfering compounds. (**b**) Cyclic voltammograms recorded for the GCE/PEDOT-CuONPs sensor in a diluted milk sample and in the presence of various amounts of H_2_O_2_. Potential scan rate: 50 mV/s.

The selectivity of the GCE/PEDOT-CuONPs sensor was assessed in chronoamperometry mode upon the successive addition of 0.25 mM H_2_O_2_ and the interfering compounds, such as KNO_3_, KNO_2_, and NaCl, respectively, each at a concentration level of 0.2 mM. The interference study was devoted to the investigation of the potential inorganic interfering species usually encountered in agri-food samples. As shown in Figure 11a, there is an obvious current response to H_2_O_2_, while the potential interfering compounds show a negligible response, indicating that the GCE/PEDOT-CuONPs sensor exhibits a high selectivity for H_2_O_2_ detection in the presence of potential interfering species.

**Figure 12:**
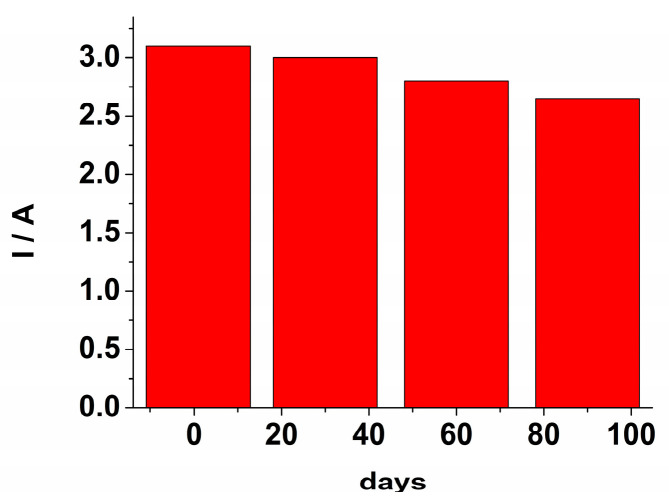
The sensor’s response towards H_2_O_2_ at various interval times over a period of 90 days.

The stability and reusability of the GCE/PEDOT-CuONPs sensor were also investigated. The sensor was kept in a refrigerator, and its response to H_2_O_2_ was checked monthly. The response of the electrochemical sensor after three months retained 85% from the initial response (see Figure 12). The steady-state response time was estimated as the time required to achieve 90% of the current response at steady-state, and a value of 8s as obtained. The repeatability, expressed as the relative standard deviation (rsd%), was estimated by measuring three times an H_2_O_2_ level of 40 μM using the same electrode. A repeatability value of 3.3% was obtained. The reproducibility (rsd%) was assessed by measuring a 40 μM H_2_O_2_ level using three different sensors, and a value of 15.4% was obtained.

The applicability of the GCE/PEDOT-CuONPs for H_2_O_2_ detection in real samples was also investigated. The sensor was applied in the analysis of milk samples using the cyclic voltammetry technique and the standard addition protocol. The use of hydrogen peroxide in the food industry for preserving the quality of various foods is a well-established approach. In this sense, the proposed sensor was tested in the analysis of milk samples to explore its potential applicability and analytical performance. Figure 11b displays the cyclic voltammograms recorded for the GCE/PEDOT-CuONPs sensor in 0.1 M NaOH containing the diluted milk sample and various added amounts of H_2_O_2_. The milk sample was diluted with electrolytes in a 1:20 volume ratio, and the corresponding cyclic voltammograms were recorded after each addition of H_2_O_2_. The redox peaks specific to CuO are visible at −0.45 V for the cathodic one and at +0.12 V for the anodic one, respectively. There is a linear increase in the cathodic peak current with the H_2_O_2_ addition, attesting to the good analytical response of the sensor toward the target analyte. From the corresponding calibration plot, the H_2_O_2_ concentration in the milk sample was computed. H_2_O_2_ was not detected in the milk sample. Furthermore, the recovery values for various H_2_O_2_ additions, from 40 to 160 μM, were in the range of 100% to 108%, demonstrating the good accuracy of the proposed sensor.

These results attest to the good analytical performance in terms of the low detection limit, wide linear response range, fast response time, anti-interference capability, repeatability, and stability of the developed GCE/PEDOT-CuONPs electrochemical sensor and point out the potential applications of the H_2_O_2_ detection in agri-food samples with a complex composition and matrix.

## 4. Conclusions

In this work, a new copper(II) oxide material, denoted as CuONPs, obtained through the polyol method was used in the development of a new modified CuONPs/GCE/PEDOT electrochemical sensor for H_2_O_2_ detection. The hydrolysis process of copper(II) acetate in 1,4-butanediol afforded, in mild reaction conditions, copper(II) oxide particles, which were rarely obtained in diol-assisted synthesis. The XRD data confirms the formation of a pure phase of monoclinic tenorite (CuO), whereas the microstructural analysis shows the formation of large aggregates of spherical nanoparticles (of ca. 8 nm). The sensor is based on a glassy carbon electrode modified by a conducting organic polymeric layer of PEDOT as a suitable matrix for CuONP catalyst. The electrochemical behavior of H_2_O_2_ at the GCE/PEDOT-CuONPs demonstrated the benefits of the CuONP material in the improved electron transfer process as well as in the overall analytical performance. The GCE/PEDOT-CuONPs sensor displayed a linear response range from 0.04 to 10 mM H_2_O_2_. The low detection limit of 8.5 μM H_2_O_2_ attests to the good capability of the CuONP-based sensor to perform a sensitive analytical detection of the analyte and a comparable performance of the proposed sensor to other sensors previously published in the literature. The proposed sensor demonstrated a good accuracy for different H_2_O_2_ additions (from 40 to 160 μM), and the recovery values were in the range of 100% to 108%.

## Figures and Tables

**Table 1:** The analytical performance comparison for previous hydrogen peroxide electrochemical sensors with the GCE/PEDOT-CuONPs sensor.

Electrode Materials	Linear Range (µM)	Detection Limit (µM)	Working Potential (V)	References
PEDOT-CuO	40–10,000	8.5	−0.4	This work
^a^ CuGa_2_O_4_	5–200	5	−0.45	[57]
^b^ SeNPs-FTO	100–20,000	79.3	−0.9	[58]
^c^ LSG-Ag	100–10,000	7.9	−0.5	[59]
Ag electrode	10–22.5 × 10^3^	6	−0.3	[60]
^d^ Fe/CV-CPE	20–1100	20	−0.15	[61]
^e^ FTS-PPy-42.1%	10–4000	5	−0.73	[62]
^f^ CuO nanosheet/Cu foil	10–20,000	10	−0.25	[48]
^g^ NC@Cu_x_O amorphous Cu_x_O with oxygen vacancies	0–500	6.7	−0.41	[63]
^h^ Nest-like CuO nanostructures	2500–300,000	440	−0.2	[25]
^i^ Hierarchical CuO nanoflowers	5–14,070	120	−0.4	[47]
^j^ SS/AuNPs	10–1000	3.97	−0.6	[64]
^k^ Cu@Pt-Pd/C	5–250	0.37	−0.10	[65]
^l^ CoPCF16	4.4–1760	0.03	−0.23	[66]
^m^ Co_3_O_4_/NiCo_2_O_4_	50–41,700	0.2578	0.3	[67]

^a^ CuGa_2_O_4_, spinel-type CuGa_2_O_4_ nanoparticle-modified glassy carbon electrode; ^b^ SeNPs-FTO, selenium nanoparticles coated on fluorine-doped tin oxide glass substrate; ^c^ LSG-Ag, laser-scribed graphene decorated with silver nanoparticles; ^d^ Fe/CV-CPE, carbon paste electrode modified with Fe-exchanged zeolitic volcanic tuff; ^e^ FTS-PPy-42.1%, composite core-shell spheres with FTS (Fe_2_O_3_/TiO_2_/SiO_2_) and a polypyrrole content of 42.1%; ^f^ CuO nanosheet/Cu foil; ^g^ NC@Cu_x_O amorphous Cu_x_O with oxygen vacancies; ^h^ nest-like CuO nanostructures; ^i^ hierarchical CuO nanoflowers; ^j^ stainless steel, gold nanoparticles; ^k^ Cu@Pt-Pd/C, core shell Cu@Pt-PD/C nanoparticles; ^l^ CoPCF16, molecular fluorinated cobalt phthalocyanine; ^m^ Co_3_O_4_/NiCo_2_O_4_ nanocomposite.

## Data Availability

Data is contained within the article.
